# Modes of Dying

**Published:** 1875-03

**Authors:** 


### MODES OF DYING.

The brain, the heart, and the lungs, may be called the three great central wheels of the living machine. Mutually dependent, any derangement in the functions of one is felt by each of the others. Should the action of one entirely cease, all would stand still. Accordingly, as one or the other is the first to feel the approach of death, it is said that death *begins* at the brain, or the lungs, or the heart.

As an example of death beginning at the brain, we mention apoplexy, in which the pressure from distended blood-vessels, or from the presence of blood from a ruptured vessel, so greatly interferes with the action of the brain as to cause death. The same result follows from the pressure of a depressed fragment of bone in a fractured skull.

Opium and other narcotic drugs act specifically on the brain; and, when taken in poisonous quantities, death begins in that organ.

In the operation of hanging, if the neck is broken, the pressure of the fractured portion of the cervical vertebra upon the *medulla oblongata*, which is but a continuation of the brain from the cranial cavity, produces death instantly. But if the neck is not broken, and the victim dies from strangulation, we have an example of death beginning at the lungs. Any obstruction of the lungs, whether the peculiar exudation of croup which fills the larynx, and is known as the *false membrane*, or any other which prevents the access of air, causes death by strangulation. In extensive pulmonary congestion, and in solidification of the lungs from inflammation, the natural, spongy tissue, is rendered impervious to air, and death begins at the lungs. Drowning, and suffocation by any means, as well as the inhalation of poisonous gas, are other examples of this mode of dying. Death *beginning at the heart* may be sudden or gradual. The opposite conditions of paralysis and spasm may be produced by the same cause, such as wounds of the heart, shock from extensive injury, as the crushing of a limb, or a violent blow on the pit of the stomach. If, from paralysis, the heart ceases to contract, and is found distended with blood. If, from spasm, the organ is powerfully contracted, its cavities are emptied and almost obliterated. Sudden death from drinking ice-water when the body is over-heated is by some supposed to be caused by "cramp in the stomach." But it is spasm, or "cramp" of the heart, which is the immediate cause of death in those cases. The violent shock of the cold liquid on the nerves of the stomach is transmitted to the heart by reflex action, and produces the powerful and fatal contraction.

Many fatal cases of sunstroke are examples of death from paralysis of the heart. Exhausted by the heat and over-exertion, the patient faints, and never revives. The heart loses its power to contract; and, in a state of complete relaxation, its cavities loaded with blood, motion ceases.

The example mentioned will serve as a type of cases of sudden death from paralysis of the heart. A person prostrated by loss of blood is suddenly raised up in bed—he falls back and is dead. The heart, weakened in common with the whole muscular system, is unable to perform the extra labor forced upon it by the change of position.

Death beginning at the heart is not always sudden. In fevers and other continued diseases, which gradually exhaust the vital powers, the action of the heart becomes by degrees irregular and feeble, until at last it flutters and dies.

For practical purposes, it is sufficient to recognize but two modes of dying, viz., Death from *asthenia*, or want of strength, and from *apnœa*, or want of breath. The common saying that a man died "from want of breath" is not always correct. His breathing apparatus may have been in perfect condition, and no lack of pure air, still he dies from want of strength,—his heart has not the strength to continue its labor, and *there* death begins. Or, he is in good strength, but some of the conditions above mentioned interfere with respiration, and he dies from *apnœa*, want of breath.

---

Brick by brick, houses are built; little by little, knowledge is gained.

### WOMAN'S LOVE.

No woman will love a man the better for being renowned or prominent. Though he be first among men, she will only be prouder, not fonder; as is often the case, she will not even be proud. But give her love, appreciation, kindness, and there is no sacrifice she would not make for his content and comfort. The man who loves her well is her hero and her king. No less a hero to her, though he is not to any other; no less a king, though his only kingdom is her heart and home.

It is a man's own fault if he is unhappy with his wife, in nine cases out of ten. It is a very exceptional woman who will not be all she can be to an attentive husband, and a more exceptional one who will not be very disagreeable if she finds herself wilfully neglected. It would be easy to hate a man who, having bound a woman to him, made no effort to make her happy; hard not to love one who was constant and tender; and when a woman loves she always strives to please. The great men of this world have often been wretched in their domestic relations, while mean and common men have been exceedingly happy. The reason is very plain. Absorbed in themselves, those who desired the world's applause were careless of the little world at home; while those who had none of this egotism strove to keep the hearts that were their own, and were happy in their tenderness.

---

Perseverance, by its daily gains, enriches a man more than fits and starts of fortune and speculation.

Every day a thread, makes a skein a year.

